# Impaired value-based decision-making in Parkinson’s disease apathy

**DOI:** 10.1093/brain/awae025

**Published:** 2024-02-02

**Authors:** William Gilmour, Graeme Mackenzie, Mathias Feile, Louise Tayler-Grint, Szabolcs Suveges, Jennifer A Macfarlane, Angus D Macleod, Vicky Marshall, Iris Q Grunwald, J Douglas Steele, Tom Gilbertson

**Affiliations:** Division of Imaging Science and Technology, Ninewells Hospital and Medical School, University of Dundee, Dundee DD1 9SY, UK; Department of Neurology, Ninewells Hospital and Medical School, Dundee DD1 9SY, UK; Division of Imaging Science and Technology, Ninewells Hospital and Medical School, University of Dundee, Dundee DD1 9SY, UK; Department of Neurology, Ninewells Hospital and Medical School, Dundee DD1 9SY, UK; Rehabilitation Psychiatry, Murray Royal Hospital, Perth PH2 7BH, UK; Rehabilitation Psychiatry, Murray Royal Hospital, Perth PH2 7BH, UK; Division of Imaging Science and Technology, Ninewells Hospital and Medical School, University of Dundee, Dundee DD1 9SY, UK; Division of Imaging Science and Technology, Ninewells Hospital and Medical School, University of Dundee, Dundee DD1 9SY, UK; Medical Physics, Ninewells Hospital and Medical School, Dundee DD1 9SY, UK; SINAPSE, University of Glasgow, Imaging Centre of Excellence, Level 2, Queen Elizabeth University Hospital, Glasgow G51 4TF, Scotland, UK; Institute of Applied Health Sciences, School of Medicine, University of Aberdeen, Foresterhill, Aberdeen AB24 2ZD, UK; Department of Neurology, Aberdeen Royal Infirmary, Foresterhill, Aberdeen AB24 2ZD, UK; Institute of Neurological Sciences, Queen Elizabeth University Hospital, Glasgow G51 4TF, UK; Division of Imaging Science and Technology, Ninewells Hospital and Medical School, University of Dundee, Dundee DD1 9SY, UK; Division of Imaging Science and Technology, Ninewells Hospital and Medical School, University of Dundee, Dundee DD1 9SY, UK; Division of Imaging Science and Technology, Ninewells Hospital and Medical School, University of Dundee, Dundee DD1 9SY, UK; Department of Neurology, Ninewells Hospital and Medical School, Dundee DD1 9SY, UK

**Keywords:** Parkinson’s disease, apathy, decision-making, computational modelling, functional MRI, reward insensitivity

## Abstract

Apathy is a common and disabling complication of Parkinson’s disease characterized by reduced goal-directed behaviour. Several studies have reported dysfunction within prefrontal cortical regions and projections from brainstem nuclei whose neuromodulators include dopamine, serotonin and noradrenaline. Work in animal and human neuroscience have confirmed contributions of these neuromodulators on aspects of motivated decision-making. Specifically, these neuromodulators have overlapping contributions to encoding the value of decisions, and influence whether to explore alternative courses of action or persist in an existing strategy to achieve a rewarding goal.

Building upon this work, we hypothesized that apathy in Parkinson’s disease should be associated with an impairment in value-based learning. Using a four-armed restless bandit reinforcement learning task, we studied decision-making in 75 volunteers; 53 patients with Parkinson’s disease, with and without clinical apathy, and 22 age-matched healthy control subjects. Patients with apathy exhibited impaired ability to choose the highest value bandit. Task performance predicted an individual patient’s apathy severity measured using the Lille Apathy Rating Scale (*R* = −0.46, *P* < 0.001). Computational modelling of the patient’s choices confirmed the apathy group made decisions that were indifferent to the learnt value of the options, consistent with previous reports of reward insensitivity. Further analysis demonstrated a shift away from exploiting the highest value option and a reduction in perseveration, which also correlated with apathy scores (*R* = −0.5, *P* < 0.001).

We went on to acquire functional MRI in 59 volunteers; a group of 19 patients with and 20 without apathy and 20 age-matched controls performing the Restless Bandit Task. Analysis of the functional MRI signal at the point of reward feedback confirmed diminished signal within ventromedial prefrontal cortex in Parkinson’s disease, which was more marked in apathy, but not predictive of their individual apathy severity. Using a model-based categorization of choice type, decisions to explore lower value bandits in the apathy group activated prefrontal cortex to a similar degree to the age-matched controls. In contrast, Parkinson’s patients without apathy demonstrated significantly increased activation across a distributed thalamo-cortical network. Enhanced activity in the thalamus predicted individual apathy severity across both patient groups and exhibited functional connectivity with dorsal anterior cingulate cortex and anterior insula.

Given that task performance in patients without apathy was no different to the age-matched control subjects, we interpret the recruitment of this network as a possible compensatory mechanism, which compensates against symptomatic manifestation of apathy in Parkinson’s disease.

## Teaser

Apathy is a common complication of Parkinson’s disease. Gilmour, Mackenzie *et al*. show that apathy can be related to a reduced ability to use information encoded in the frontal lobes about the outcomes of an action. Patients without apathy use an additional circuit to compensate for this impairment and prevent demotivation.

See Heron *et al*. (https://doi.org/10.1093/brain/awae084) for a scientific commentary on this article.

## Introduction

Apathy is a debilitating and poorly understood syndrome characterized by a reduction in goal-directed behaviour. In neurodegenerative conditions including Parkinson’s disease (PD) it is estimated to affect 30%–70% of patients.^1-5^ In contrast to the defining motor symptoms of this condition, apathy is a greater predictor of poor quality of life.^6^ Apathy is associated with a higher likelihood of developing dementia^7^ and presents a clinical challenge, due to its resistance to dopamine replacement or any other treatments.^8^ Importantly, apathy is understood to be a distinct process and a direct consequence of neurodegeneration, independent from co-morbid mood disorder or a secondary consequence of physical disability of the disease.^2,9^ Although the burden of apathy on patients with neurodegenerative disease is increasingly recognized clinically,^10^ limited progress has been made in understanding the neural circuit mechanisms.^11^ Understanding these is crucial to developing novel treatments, as different dysfunctional neural process are likely to contribute to the manifestation of apathy in different clinical populations.^12^

Neuroimaging studies of PD patients with apathy consistently identify structural or functional imaging abnormalities within prefrontal cortical circuits and their reciprocally connected sub-cortical nuclei of the basal ganglia.^13^ These abnormalities include the orbitofrontal cortex (OFC),^14,15^ ventromedial prefrontal cortex,^16-18^ anterior cingulate cortex (ACC),^15,18^ caudate and ventral striatum.^13,15,16^ Abnormalities of brain volume and functional activation have also been localized to the same areas in patients with apathy with different neurodegenerative conditions^19,20^ supporting a transdiagnostic anatomical basis.

The function of this fronto-striatal circuit in decision-making has been refined by decades of cognitive neuroscience research and include discrete contributions to evaluation of effort costs, choice arbitration and the encoding of the value of actions and sensory stimuli.^21-24^ Based on these functions and reviewing neuroimaging studies of apathy, Le Heron *et al*.^19^ proposed that apathy arises from dysfunction within a fronto-striatal circuit that mediates any of three key elements of motivated behaviour: (i) deciding whether to act; (ii) persisting with an action; and (iii) learning, through outcome monitoring, whether a behaviour was worth performing.

In support of a higher threshold for (i) deciding whether to act; when faced with the decision to exert effort for a monetary reward, apathetic patients tend to reject more offers compared to non-apathetic counterparts.^25^ This behaviour is not due to heightened sensitivity to effort costs, but rather points to diminished incentivization by rewarding outcomes, a characteristic feature of apathy.^25^ This interpretation aligns with the observations of reward insensitivity as a general feature of apathy in PD, corroborated by diminished pupillary response,^26^ decreased ventral striatal activation^16^ and feedback-related negativity (FRN) signals to rewarding stimuli.^27^

Reward outcome encoding has long been thought of as a function of dopamine projections to the frontal-striatal circuit.^28,29^ However, outside of specific contexts (such as postoperative apathy following deep brain stimulation^30^), dopamine replacement does not restore the motivational deficit in the decision to act in apathetic patients.^25^ Furthermore, the results of dopamine replacement treatment strategies have been mixed^31^; there is no clear relationship between apathy severity and dopaminergic medication dose.^3^ Neuromodulators, including serotonin and noradrenaline, have overlapping functions with dopamine in encoding an actions value and determining how the brain uses this information to guide future decisions.^32-40^ Deficiencies in serotonergic^41,42^ and noradrenergic neurotransmission^43-45^ correlate with the severity of apathy in PD. Therefore, a unifying explanation for PD apathy would be either a failure to encode the value of actions, or an impairment in using this information, to learn that an action is worth performing and motivate behaviour.^19^

Given this context, we tested the hypothesis that apathy in PD is characterized by a decision-making signature reflecting a primary failure of outcome monitoring and/or value-based choice.^19^ To test this, we chose a classical reinforcement learning task, the four-armed restless bandit,^46,47^ as its performance relies on the ability to constantly update both the short and longer-term outcomes of each decision. Owing to the dynamic and constantly varying payout of each of the ‘bandits’, performance relies on adaptive behaviour, which balances exploitation with exploration.^47-49^ Using a computational model-based functional MRI (fMRI) design^50,51^ we aimed to identify regions of the prefrontal cortex which underpin apathy in PD.

## Materials and methods

### Ethics

Seventy-seven participants were recruited for the study, which was approved by the local ethics committee (North East Scotland 21/ES/0035). Written consent was obtained from all participants in accordance with the Declaration of Helsinki.

### Patient group

Fifty-five patients with a clinical diagnosis of idiopathic PD were recruited from movement disorders clinics in NHS Tayside, Grampian and Greater Glasgow and Clyde, UK. Diagnosis was confirmed by a consultant neurologist (T.G., V.M., A.D.M.) guided by UK Brain Bank criteria.

### Control group

Twenty-two age- and sex-matched healthy control subjects were recruited via the SHARE health informatics register (https://www.registerforshare.org/). Healthy controls were screened for a history of significant neurological or psychiatric conditions.

### Exclusion criteria

Patients were excluded if they had a diagnosis of PD dementia or any other co-morbid neuropsychiatric diagnosis, including major depressive disorder. PD patients on anti-depressant therapy in remission from depression were included in the study but could not be under active treatment by a consultant psychiatrist. No patient was receiving antipsychotic medication. Two patients were excluded as their Montreal Cognitive Assessment score was within the abnormal range (MoCA < 24).

### Procedure

Participants performed two sessions. The first, ‘out-of-scanner’ session, involved performing the Restless Bandit Task on a laptop, while in the second ‘in-scanner’ session the task performed during fMRI image acquisition. All assessments and tasks were performed with the patients on their usual Parkinson’s medications. If patients had no contra-indications for MRI scanning (e.g. contra-indicated metal implantation, claustrophobia or significant dyskinesia that could lead to image motion artefacts), they underwent both sessions on the same day.

### Clinical rating scales

Apathy was assessed using the Lille Apathy Rating Scale (LARS) a questionnaire specifically validated for assessment of PD. LARS scores range from −36 to +36 with scores > −22 considered apathetic.^52^ PD severity was assessed using part III of the Movement Disorders Society Unified Parkinson’s Disease Rating Scale (UPDRS)^53^ in the ON medication state. Mood and anxiety scores were assessed using the Hospital Anxiety and Depression Scale (HADS-A and D). Cognitive screening was performed using the MoCA. Participant demographics are in Table 1. LARS factorial subscores are provided in Supplementary Table 1.

**Table 1:** Patient demographics and clinical details

	Healthy controls	Parkinson’s disease	Control versus PD (*P*-value)	PD-apathy (LARS > −22)	PD no-apathy (LARS < −22)	PD-apathy versus PD no-apathy (*P*-value)
*n*	22	53	n/a	25	28	n/a
Age	62.8 ± 8.5	62.9 ± 8.9	0.99	61.6 ± 11.3	63.8 ± 6.4	0.39
Gender, male:female	15:7	37:14	0.78^a^	20:3	17:11	0.06^a^
Disease duration (years)	n/a	6.6 ± 3.7	n/a	6.7 ± 3.6	6.6 ± 3.9	0.91
Apathy (LARS)	−28.1 ± 3.6	−21.7 ± 8.1	**<0.001**	−14.0 ± 5.2	−28.0 ± 2.9	**<0**.**001**
UPDRS-III	n/a	30.3 ± 8.7	n/a	31.9 ± 8.6	28.7 ± 8.7	0.25
Levodopa equivalent dose (mg/24 h)	n/a	643 ± 368	n/a	608 ± 388	673 ± 354	0.55
Dopamine agonist (number of patients)	n/a	24	n/a	11	13	0.99^a^
Dopamine agonist (levodopa equivalent dose mg/24 h)	n/a	199 ± 96	n/a	174 ± 80	219 ± 110	0.57
HADS-D	2.3 ± 2.4	5.5 ± 3.7	**<0.001**	8.5 ± 2.6	3.0 ± 2.4	**<0**.**001**
HADS-A	4.9 ± 2.8	6.7 ± 4.1	0.07	8.1 ± 4.2	5.5 ± 3.7	**0**.**02**
MoCA	27.4 ± 2.0	27.6 ± 1.7	0.67	27.1 ± 2.0	28.0 ± 1.24	0.07

Values are expressed as mean ± standard deviation. HADS = Hospital Anxiety and Depression Rating Scale; LARS = Lille Apathy Rating Scale; MoCA = Montreal Cognitive Examination; n/a = not applicable; PD = Parkinson’s disease; UPDRS = Unified Parkinson’s Disease Rating Scale. Two-tailed unpaired *t*-test significant differences are highlighted in bold.

^a^Fisher’s exact test.

### Experimental design

Participants performed the Restless Bandit Task.^46,47^ Subjects were given written instructions on how to perform the task, were told that with each trial they could win between 0 and 100 points and agreed to maximize outcome points.

**Figure 1:**
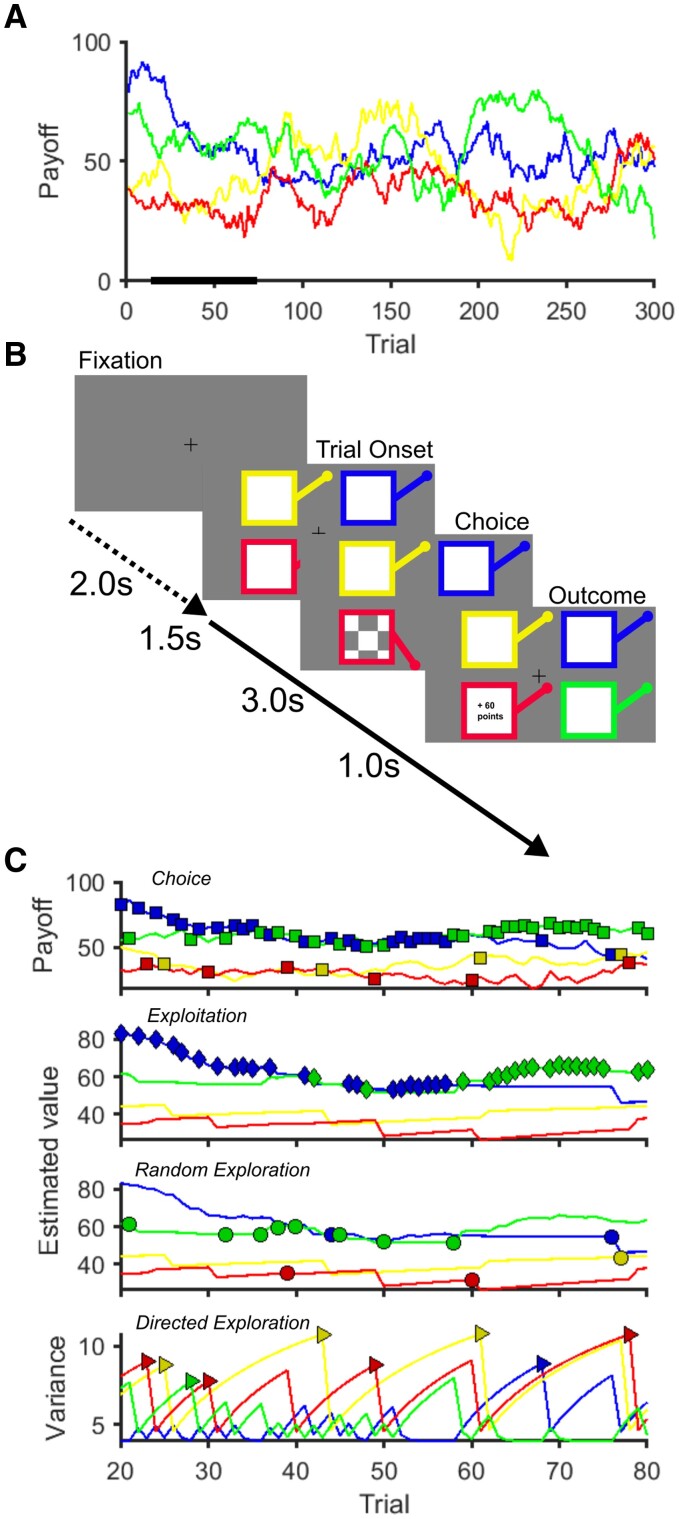
**Restless Bandit Task.** (**A**) Example of the underlying payout (reward) structure across the 300 trials of the task for each of the four bandits. The payout varied from one trial to the next by a Gaussian walk. (**B**) Each trial has a fixed trial length of 6 s with a variable inter-trial interval designated by the time between the fixation cross and the onset of the trial (mean 2 s). At trial onset, four coloured squares (bandits) were presented. The participant selected one bandit within 1.5 s, which was then highlighted by the bandit lever depressing and a chequer board appearing (choice screen). After a 3 s delay the, the outcome of the choice, as number of ‘points’ won, was displayed for 1 s. In trials where the participant failed to make a choice within 1.5 s, the choice screen was replaced by a large red cross (not shown) signifying a missed trial. (**C**) An example of a single subject’s choices in the task (*top*) fitted to a reinforcement learning model used to estimate the latent neural encoding of each bandits estimated value and uncertainty (variance). This model allows choices to be categorized into one of three decision types depending upon whether these were made to the bandit with the highest estimated value (exploitative choice) or to one of the three lower valued options (random exploration). Directed exploration choices (*bottom*) are to a lower valued bandit, which has least recently been selected and there is most uncertainty as to its true value.

Each trial started with presentation of four different coloured squares with all four bandit’s levers in the upright position representing the four choice options (Fig. 1). Participants made their selection using a four key mini-keyboard (Ecarke-EU) with the colour of each button corresponding to a square presented on the computer monitor corresponding to each of the ‘bandits’. If a button press was not made within a 1.5-s response deadline, a large red ‘X’ was displayed for 4.2 s at the centre of the screen. These trials were designated as missed trials and no outcome feedback was provided. For choices made within the response deadline, the chosen bandit was highlighted with its lever shown depressed and a checker-board pattern appeared at the centre of the bandit’s square. After a 3-s waiting time, this pattern was replaced by the outcome number of points earned on that trial in the centre of the chosen bandit’s square for 1 s. Then the bandit image disappeared and was replaced by a fixation cross until 6 s after the trial onset, followed by a jittered inter-trial interval [Poisson distribution, mean: 2 s (0–5 s)] before the next trial was started. The payout (outcome) schedule of each of the four bandit choices varied according to a decaying Gaussian random walk. We used two instantiations from Daw *et al*.^46^ for the two experimental sessions and the order of sessions was the same for all subjects.

During the ‘in-scanner’ session participants made responses using an MRI-conditional button box. During this session, Restless Bandit Task images were projected onto a screen visible to the patient inside the MRI scanner. The task was implemented using MATLAB (R2021; MathWorks, Natick, MA) running Psychophysics Toolbox (version 3.0.12).^54^

Of the 53 patients who participated in the out-of-scanner session, 40 (20 with apathy, 20 without) also agreed to an ‘in-scanner’ session, along with 20 of the healthy controls. One patient in the apathy group was unable to perform the task in the scanner and was excluded from final analysis.

The task consisted of 300 trials. Participants were given short breaks after every 75 trials to improve concentration and task engagement.

### Analysis of behavioural performance

Model-free metrics of behavioural performance in the task included best bandit choice probability, decision time and the probability of missing a trial. Decision time was defined as the time between the four bandits being presented and the participants’ button press on that trial. Where a measure of behavioural performance is expressed as a probability, this was achieved by dividing by the total number of trials correctly executed within response deadline, either in a 50-trial block, or by dividing this by the total number of responses made across the whole task.

### Computational modelling of decision-making

To understand the decision-making process further we fitted eight computational model variations to the experimental choices from the bandit task.

Each model variant used one of two learning rules (Delta rule, or Bayesian learner) combined with one of four choice rules: (i) SoftMax (SM); (ii) SoftMax with exploration bonus (SME); (iii) SoftMax with perseveration bonus (SMP); and (iv) SoftMax with exploration and perseveration bonuses (SMEP). By modelling the latent neural estimate of each bandit’s value, and the brain’s imperfect knowledge about this (the estimated variance), we were able to classify each decision made into one of three types (Fig. 1C): (i) an exploitative choice to the bandit with the highest estimated value; (ii) directed exploration, to a lower valued option, which has least recently been chosen and whose value there is least confidence about; and (iii) random exploration to a lower valued bandit, regardless of current knowledge about its payout.^55,56^

Posterior distributions were estimated for each subject for each of the model’s free parameters. These included α (the learning rate in the Delta rule), β (the inverse temperature parameter), ϕ (the exploration bonus) and ρ (the perseveration bonus). β is also commonly referred to as the reward sensitivity parameter as it multiplies the estimated value of the bandits. Larger values of β index an ‘exploitative’ choice policy, with lower values reflecting an ‘exploratory’ strategy and more specifically random exploration.^47^ By multiplying the estimated variance of the bandits, scales proportionately with the amount of directed exploration.^47,57^ ρ was included in the SMEP and SMP choice rules, as modelling perseveration (i.e. choosing the same bandit on two consecutive trials irrespective of their estimated value), improves model fit in previous studies using the Restless Bandit Task.^46,47^ Details of the model and fitting procedure are provided in the Supplementary material.

### Functional MRI methods

For each participant, functional whole-brain images acquired with a 3 T Siemens Prisma Fit scanner using an echo planar imaging sequence with the following parameters: repetition time/echo time = 2500/26 ms, flip angle = 90°, field of view = 224 mm, matrix = 64 × 64, 37 slices, voxel size 3.5 × 3.5 × 3.5 mm, slice gap = 0.5 mm.

The fMRI analysis was performed on three distinct participant groups: healthy controls, patients diagnosed with PD who exhibited symptoms of apathy, and patients with PD without apathy symptoms. We conducted a first-level analysis (detailed in the Supplementary material) by creating a general linear model (GLM) for each participant. This was done separately for each group across the four blocks of the in-scanner task.

Using a second-level random effects approach, the subject- and group-specific contrast images for each first-level regressor were submitted to a full factorial model in SPM12. For each contrast-specific second-level analysis, a T-contrast image was generated and tested for the main effect of that contrast over all subjects for each of the groups.

We performed whole-brain analyses of both activation within groups and between group contrasts and report activations and between group contrasts surviving cluster-level family-wise error (FWE) correction at *P* < 0.05 (indicated with *P*_cluster FWE WB_), corresponding to a simultaneous requirement for a voxel threshold of *P* < 0.001 and a minimum cluster size of 10 voxels. Region of interest (ROI) analyses were performed using a 10 mm sphere centred on a peak voxel of interest (indicated with *P*_peak FWE SVC_).

### Statistical analysis

We used a mixed-design ANOVA with a fixed effect (between subject) of group with three levels (PD-apathy, PD-no apathy, healthy control) and a random effect (within subject) variable of within task block with (six, 50-trial blocks). All results are reported as mean values ± standard error of the mean (SEM).

## Results

### Patients with apathy are less likely to choose the best bandit

We acquired choice behaviour from 53 PD patients (25 with apathy, ‘PD-apathy’, and 28 without, ‘PD-no apathy’) as well as 22 age- and sex-matched healthy control subjects (‘HC’), performing the Restless Bandit Task (Fig. 1). Patients with and without apathy had comparable levels of motor disability and medication status (Table 1).

**Figure 2:**
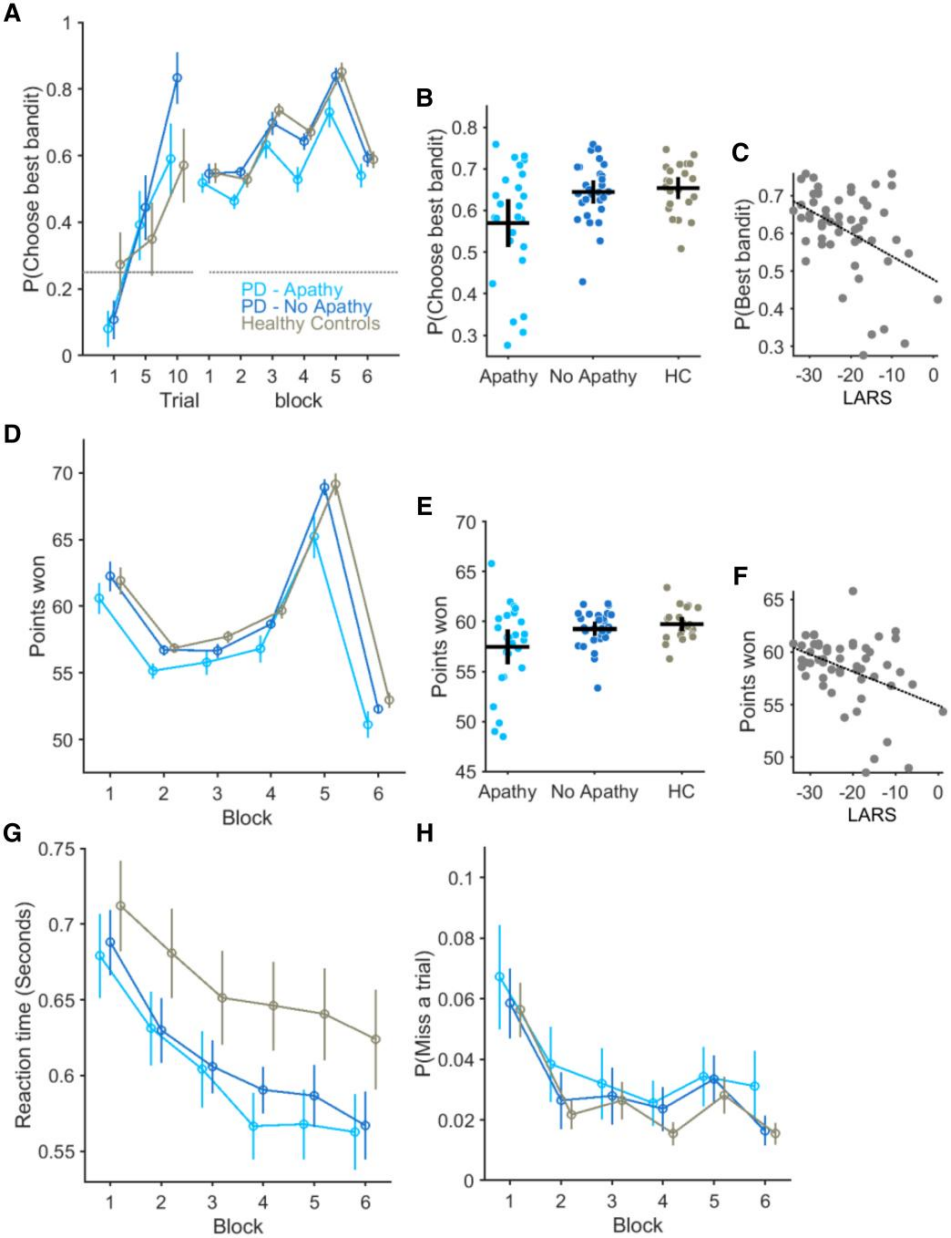
**Bandit performance and relationship with apathy severity.** The average probability of choosing the bandit with the highest payout, *P*(Choose best bandit) is plotted in the three groups of participants. (**A**) The increase in average values of best bandit choice plotted in Trials 1, 5 and 10 confirm learning in all three groups from an initial random choice to above chance levels (horizontal dashed line). Best choice performance across six 50-trial blocks of the task was reduced in the PD-apathy group. Vertical lines = standard error of the mean (SEM). (**B**) Each circle represents average best bandit choice probability across the task for an individual subject with the horizontal and vertical bar represents the group mean and 95% confidence limits. (**C**) Apathy severity, measured by increasing LARS score (more positive values represent higher levels of apathy) correlated with individuals’ ability to choose the best bandit (rho = −0.43, *P* = 0.001). Performance in the task, as measured by the number of points won, also differed between groups and predicted apathy status (rho = −0.39 *P* = 0.003) in PD (**D**–**F**). Each group’s average reaction time over the six task bins in **G** and likelihood of not making a response (**H**) (missed trial) was not affected by apathy. HC = healthy controls; LARS = Lille Apathy Rating Scale; PD = Parkinson’s disease.

In the out-of-scanner session, patients with PD-apathy learned to choose the best of the four bandits above chance levels [*P*(Best bandit): 0.54 ± 0.04] but were less likely to choose this compared to the PD no-apathy [*P*(Best bandit): 0.64 ± 0.03] or controls [*P*(Best bandit): 0.65 ± 0.03] groups; main effect of group *F*(2,344) = 5.54, *P* = 0.005 (Fig. 2A and B). The probability of choosing the best bandit correlated with each patient’s apathy severity (Fig. 2C), as indexed by their total LARS score (rho = −0.43, *n* = 56, *P* = 0.001). The average number of points won during the task was significantly lower in the PD-apathy (57.4 ± 0.8) compared to the PD no-apathy (59.4 ± 0.3) and controls (59.7 ± 0.3), main effect of group *F*(2,344) = 4.35, *P* = 0.01 (Fig. 2D and E) and correlated with their total LARS score (rho = −0.39 *P* = 0.003, Fig. 2F). To ensure that this result could not be explained by a non-specific cognitive effect, we included the individual MoCA score as a covariate in this analysis. Neither the *P*(Best bandit), *F*(1,344) = 1.43, *P* = 0.2, nor difference in the points won between the groups, *F*(1,344) = 0.61, *P* = 0.43, could be explained by differences in cognition.

Impaired best bandit choice in the task could not be clearly explained by diminished engagement in the task, as the decrease in decision time seen across blocks in both healthy controls and PD no-apathy groups was also observed in the PD-apathy group [main effect of block *F*(5,344) = 26, *P* < 0.001, Block × Group interaction *F*(10,3440344) = 0.48, *P* = 0.75, Fig. 2G]. There was no significant difference in average decision times between groups over the course of the task (decision time: PD-apathy, 0.60 ± 0.02 s, PD-no apathy 0.61 ± 0.02 and controls 0.65 ± 0.03) or effect of group on decision time [*F*(2,344) = 1.8921, *P* = 0.15]. Moreover, the apathetic patients did not miss significantly more trials than patients without apathy or healthy controls (Fig. 2H) [probability of missing a trial: PD-apathy 0.03 ± 0.01, PD-no apathy 0.03 ± 0.01, controls 0.02 ± 0.006, main effect of group *F*(2,344) = 0.5, *P* = 0.6]. We reproduced the same behaviour in the in-scanner session (Supplementary material and Supplementary Fig. 1).

### Explore-exploit trade-off predicts individual apathy severity

This analysis confirmed that PD-apathy correlated with the ability to monitor the outcome of a fluctuating payout requiring identification of the most rewarding choice. We further hypothesized that this could arise from three mutually exclusive mechanisms.

First, patients with apathy might use a perseverative strategy that minimizes cognitive effort, by making choices irrespective of the perceived value of an option.^58^ Second, apathetic patients may employ an overtly greedy choice strategy. Whilst this may initially seem advantageous, it reduces the information gained from non-greedy, exploratory choices resulting in poorer decision flexibility.^55,56^ Finally, if the neural representation of decision value is degraded (or encoded but the information disregarded), behaviour should be characterized by choice policy of heightened exploration, which reflects heightened uncertainty, about which of the task robustly, model parameters could still be recovered (Supplementary Fig. 2B–D) from synthetic choice data generated from simulated choices. These also overlapped with the experimental choices from each group (Supplementary Fig. 3).

By modelling the value of each of the four options throughout the task, we were able to categorize each choice into three categories: exploitative, directed exploratory, and random exploratory (RE) choices (Fig. 1C).

**Figure 3:**
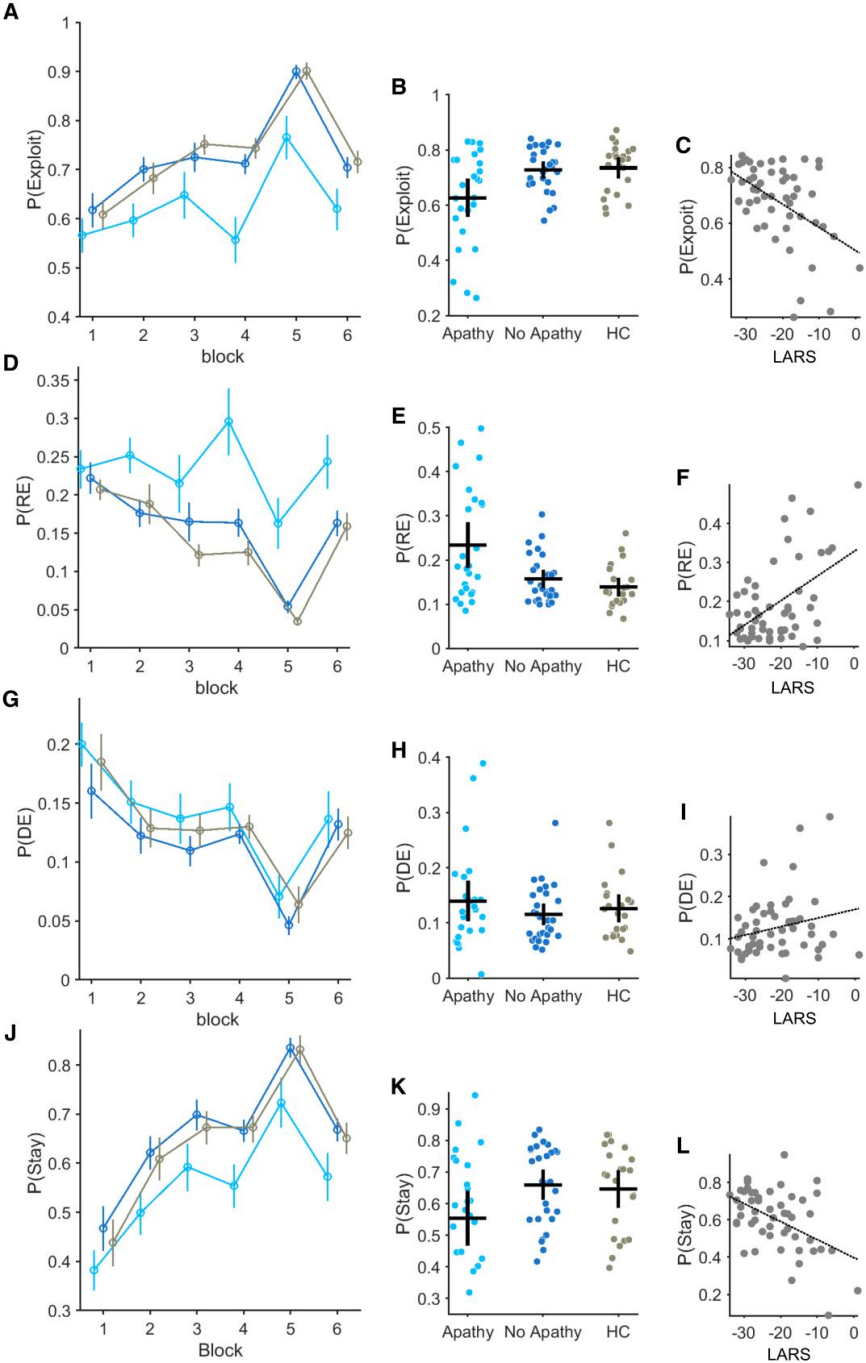
**Decision types between groups across the task and relationship with apathy severity.** (**A**–**C**) Probability of making an exploit *P*(Exploit) choice plotted across six 50-trial bins for each group tested (**A**). Error bars represent standard error of the mean (SEM). (**B**) Individual *P*(Exploit) across the whole task is represented by each circle. Group average and SEM is illustrated by the vertical and horizontal bars. Correlation between *P*(Exploit) and apathy severity (increasing LARS score) in **C** rho = −0.50, *P* < 0.001. The same analysis applied to the probability of making a random exploratory *P*(Explore) choice (**D** and **E**) and relationship with individual apathy severity (**F**) rho = 0.47, *P* < 0.001. *P*(DE) is the probability of making a directed exploratory choice (**G**–**I**) and *P*(Stay) the same choice on two consecutive trials (**J** and **K**). (**L**) Correlation between *P*(Stay) and apathy severity, rho = −0.47, *P* < 0.001. HC = healthy controls; LARS = Lille Apathy Rating Scale.

Patients with PD-apathy made a significantly higher proportion of random exploratory choices *P*(RE) than the PD-no apathy and healthy control groups (Fig. 3D–F): *P*(RE) in the PD-apathy group = 0.23 ± 0.02, PD-no apathy = 0.15 ± 0.010 and controls = 0.13 ± 0.009 [main effect of group *F*(2,344) = 8.69, *P* < 0.001]. PD-apathy patients made fewer exploitative choices through the task than non-apathetic PD patients (Fig. 3A and B) and healthy controls. *P*(Exploit): PD-apathy = 0.62 ± 0.03, PD-no apathy = 0.72 ± 0.015, controls = 0.73 ± 0.018 [main effect of group *F*(2,344) = 6.31, *P* = 0.002]. The proportion of directed exploratory choices did not differ between groups: *P*(DE); PD-apathy = 0.13 ± 0.018, PD-no apathy = 0.11 ± 0.009, controls = 0.13 ± 0.01 [main effect of group *F*(2,344) = 0.86, *P* = 0.42].

The proportion of both exploratory and exploitative choices correlated with the severity of apathy in individual patients, with *P*(RE) versus LARS recording rho(52) = 0.47, *P* < 0.001, and *P*(Exploit) versus LARS recording rho(52) = −0.50, *P* < 0.001 (Fig. 3C and F). The proportion of perseverative choices was also lower in the PD-apathy group, *P*(Stay) PD-apathy = 0.55 ± 0.04, PD-no apathy = 0.65 ± 0.036, controls = 0.64 ± 0.04 [main effect of group *F*(2,344) = 3.22, *P* = 0.04] (Fig. 3J and K) and correlated with the LARS score, rho(52) = −0.47, *P* < 0.001 (Fig. 3L).

This different decision signature between the patient groups was also reflected in the model parameter estimates. Consistent with reduced exploitation in PD-apathy group, β values were significantly lower: posterior difference in mean (Contrast = PD-apathy − PD-no apathy), M_diff_ = −0.02 (−0.028, −0.015). The exploration ϕ and perseveration bonuses, ρ, which govern the proportion of directed exploration and perseverative choices, were also lower than the PD-no apathy group: ϕ M_diff_ = −0.40 (−0.69, −0.12), ρ M_diff_ = −3.57 (−4.88, −2.27) (Supplementary Fig. 4B, E and H and Supplementary Table 2). Apathy severity correlated with the individual subjects parameter estimates, β rho(52) = −0.41, *P* = 0.002, ρ rho(52) = −0.41, *P* = 0.002 but not the exploration bonus ϕ (Supplementary Fig. 4C and I).

### Functional MRI signal of outcome encoding is blunted in apathy

Our analysis of the brain imaging data was motivated by two explanations for our apathetic patients’ behaviour. Could a failure to monitor the outcome of their actions, reflected in a shift from exploitation to exploration, be driven by a pure disorder of encoding the outcome of their decisions in the task? If the precision of the outcome’s value signal is degraded,^44^ exploration is likely to be a passive, secondary consequence of greater decision noise.^56^ Alternatively, an inability to exploit knowledge gained from learning the value of each option could arise from a failure of using this at the point at which the decision is being made.

**Figure 4:**
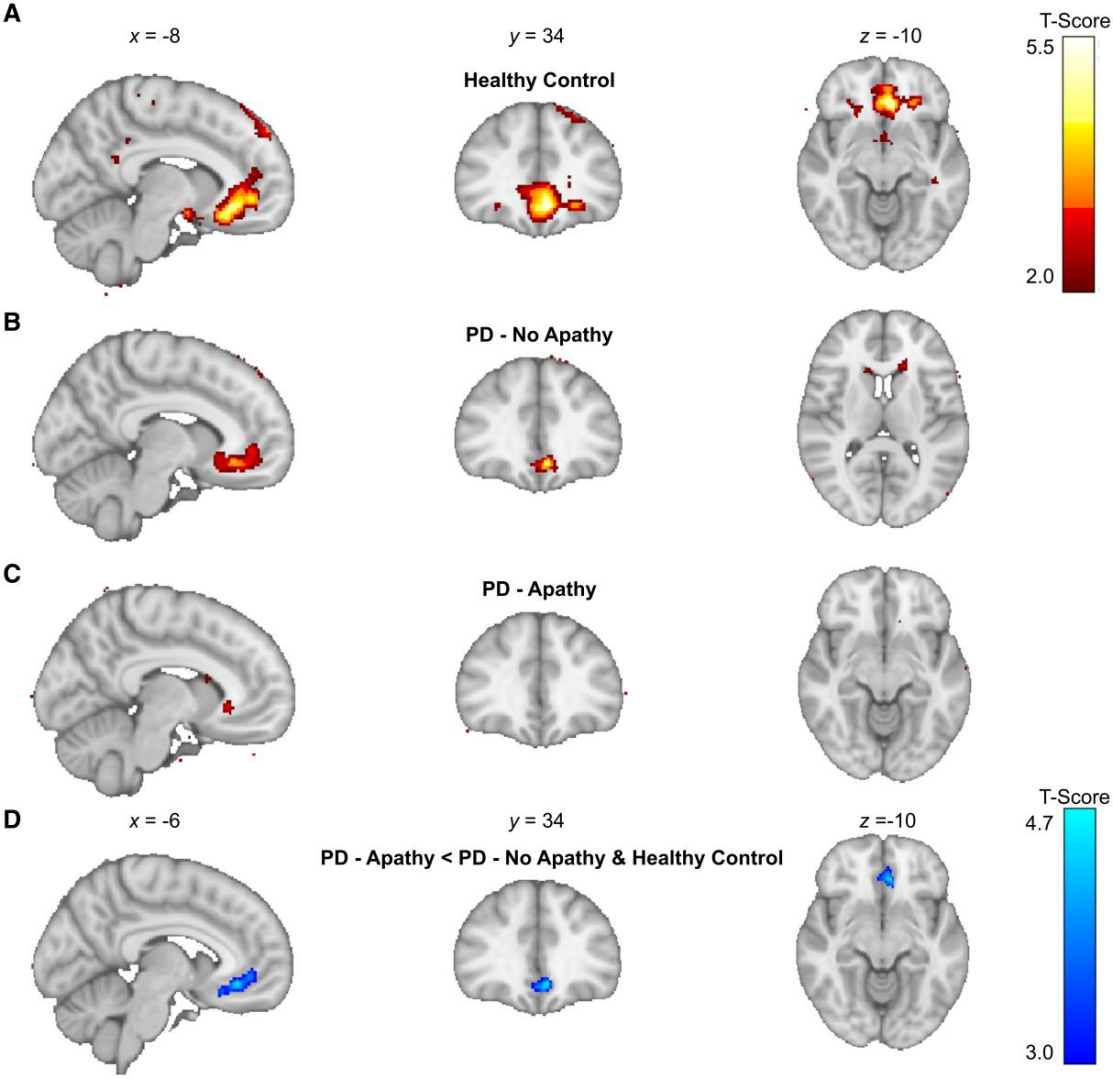
**Functional MRI BOLD correlates of the outcome signal in PD-apathy and non-apathetic groups.** (**A**) Peak activations in healthy control (HC) group at the point of feedback of the choice outcome (pay-off) time demonstrated a significant cluster in left ventromedial prefrontal cortex (vmPFC) after family-wise error (FWE) whole brain correction at *P* < 0.05 (*T* = 5.25, *P*_cluster FWE WB_ < 0.001). (**B**) Activations in PD-no apathy group did not survive correction at a whole brain level but were present in vmPFC when analysed with small volume correction using a region of interest analysis centred on the peak activation in the healthy control group (*T* = 4.51, *P*_peak FWE SVC_ = 0.004). No significant clusters activity survived whole brain or small volume correction in the PD-apathy group (**C**). Contrast analysis between groups did not demonstrate any difference in outcome signal activations between the PD-apathy and PD-no apathy groups (result not illustrated). However, combining the PD-no apathy and healthy control groups confirmed a significant reduction in the outcome signal in left vmPFC in the PD-apathy patients (**D**) (T = 4.60, *P*_cluster FWE WB_ = 0.01). BOLD = blood oxygen level-dependent; PD = Parkinson’s disease.

We proceeded to analyse the brain activity at the point in each trial at which the outcome was received and looked at the fMRI signal correlation with this payout on each trial. Replicating previous studies in healthy controls,^46,47,59,60^ we identified activity in the ventromedial prefrontal cortex (vmPFC) in our age matched healthy control group [left vmPFC: peak voxel: *x, y, z* = (−6, 34, −8), *T* = 5.25, *P*_cluster FWE WB_ < 0.001]. However, neither PD patient groups (PD-apathy and PD-no apathy) demonstrated clusters surviving whole brain correction (Fig. 4 andSupplementary Table 5).

Using an ROI analysis with small volume correction, using the peak vmPFC activations from our healthy control group, we confirmed vmPFC activation in the PD-no apathy group [left vmPFC peak voxel: (−6, 34, −10), *T* = 4.51, *P*_peak FWE SVC_ = 0.004], which was not present using the same approach in the PD-apathy group. However, contrast analysis (PD-apathy < PD-no apathy), using the same ROI, did not confirm significant reductions in the outcome signal between groups. Combining the PD-no apathy and healthy control group in a contrast analysis with the PD-apathy (PD-apathy < PD-no apathy and controls) did reveal significant reductions in outcome signal including within left vmPFC [peak voxel: (−6,34, −10), *T* = 4.60, *P*_cluster FWE WB_ = 0.01] (Fig. 4D). We also performed regression analysis of the individual LARS score in both PD groups with the payout signal at the outcome time. No significant clusters survived whole brain correction. Therefore, despite a reduction in this signal in PD-apathy patients, a difference in the encoding of the outcome signal could not provide a singular explanation for their level of apathy, or equally a shift away from exploitation to exploration in the apathetic group.

Analysis of the fMRI correlate of the reward prediction error (RPE) (Supplementary Table 9 and Supplementary Fig. 7) confirmed no between-group differences that could explain the task behaviour or apathetic status.

### Individual apathy severity correlates with cortico-thalamic activation at the decision time

**Figure 5:**
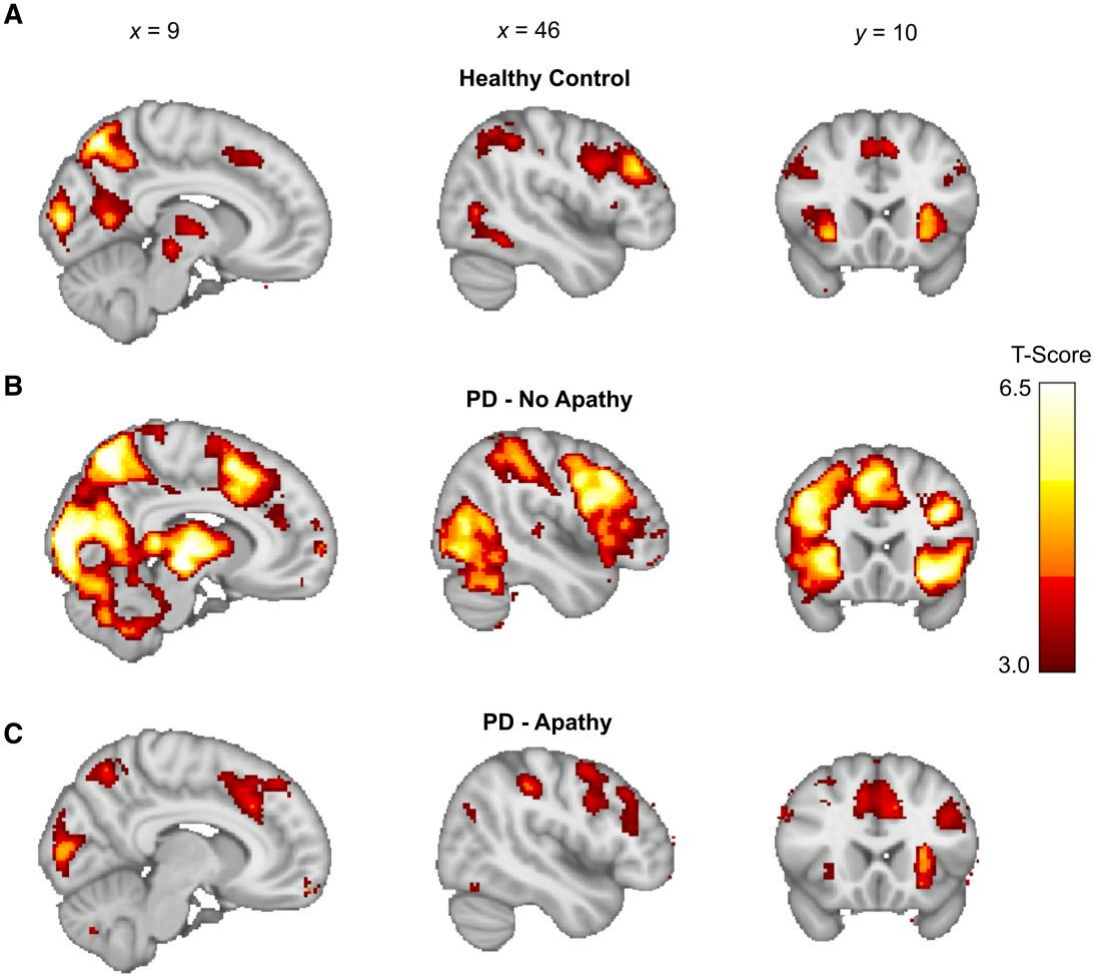
**Functional MRI BOLD signal correlates at the decision time during exploratory choices.** (**A**) Peak activations in healthy control group at the point of decision-making during exploratory choice activated parieto-occipital, thalamic and both medial (preSMA/dACC), lateral prefrontal (DLPFC) and bilateral anterior insula regions. Corresponding activation in PD-no apathy (**B**) and PD-apathy (**C**) groups. Significant clusters were defined as those surviving family-wise error whole brain correction at *P* < 0.05 (Supplementary Table 4). BOLD = blood oxygen level-dependent; PD = Parkinson’s disease.

Next, we considered brain activity at the decision-making events, focusing on distinguishing activations between exploratory and exploitative choices at trial onset (Supplementary Figs 5 and 6 and Supplementary Table 4 for in-scanner model fitting). Our findings were largely consistent with previous research, with the brain demonstrating distinct activity patterns for each choice type. In the healthy control group, exploratory trials were characterized by recruitment of occipito-parietal regions, including the calcarine cortex (CC), intraparietal sulcus (IPS) and superior parietal lobules (SPL) [peak voxel right CC: (16, −88, 10), *T* = 7.57, *P*_cluster FWE WB_ < 0.001]. This activity was in conjunction with activations observed in areas previously linked with exploration: the bilateral thalamus/midbrain (TH), anterior insula (AI), middle frontal gyrus (MFG) and supplementary motor area preSMA/dorsal anterior cingulate gyrus (dACC) [peak voxels in right TH: (8, −24, −4), *T* = 4.61, *P*_cluster FWE WB_ < 0.001, right AI: (30, 24, −4) , *T* = 5.50, *P*_cluster FWE WB_ < 0.001, right MFG: (−42, 8, 32) , *T* = 5.55, *P*_cluster FWE WB_ < 0.001, Left preSMA/dACC: (−8, 16, 46) , *T* = 5.06, *P*_cluster FWE WB_ < 0.001] (Fig. 5A and Supplementary Table 6).^46,47,59,60^

In contrast, exploit decisions in the healthy control group activated more limited regions consisting of superior frontal gyrus (SFG) and vmPFC/subgenual cingulate [peak voxels left SFG: (−18, 52, 22), *T* = 4.63 *P*_cluster FWE WB_ < 0.003; left vmPFC: (−18, 36, 4), *T* = 4.13 *P*_cluster FWE WB_ < 0.001] (Supplementary Fig. 6). No significant clusters survived whole brain correction during exploit trials in either the PD-apathy or PD-no apathy groups (Supplementary Table 7) and there was no significant difference in the activations between these groups.

The same clusters of activity during explore choices were noted in both the PD-apathy (Fig. 5C) and PD-no apathy (Fig. 5B) groups within MFG, dACC, left frontal pole and posterior occipito-parietal regions including CC, IPS, SPL and frontal pole (Supplementary Table 6). Decision to explore activations were generally more marked in PD-no apathy group than in both the healthy control and PD-apathy groups.

Comparing activations between groups, using a second level contrast (PD-no apathy > controls), confirmed additional recruitment of pre/post central gyrus (PCG), cerebellum, inferior frontal gyrus (IFG) and frontal pole [peak voxels PCG: (0, −3 068), *T* = 5.52, *P*_cluster FWE WB_ < 0.001, left cerebellum: (−26, −52, −42), *T* = 4.28, *P*_cluster FWE WB_ < 0.001, IFG: (−32, −26,14), *T* = 4.35, *P*_cluster FWE WB_ < 0.001 and frontal pole but not in TH, MFG, AI or preSMA/dACC] (Supplementary Table 8).

Further contrast analysis between the PD-apathy and healthy control groups (PD-apathy < controls) signal was comparable levels to those of healthy controls as no significant clusters were above threshold following whole brain cluster level correction (Supplementary Table 8).

In the PD-apathy group, exploratory choices were associated with intact activity across the same bilateral prefrontal (MFG), preSMA/dACC and anterior insula regions as non-apathetic participants. However, there was no comparable activation of the thalamic/midbrain and occipitoparietal regions seen during exploratory choices in the healthy controls and non-apathetic PD groups.

**Figure 6:**
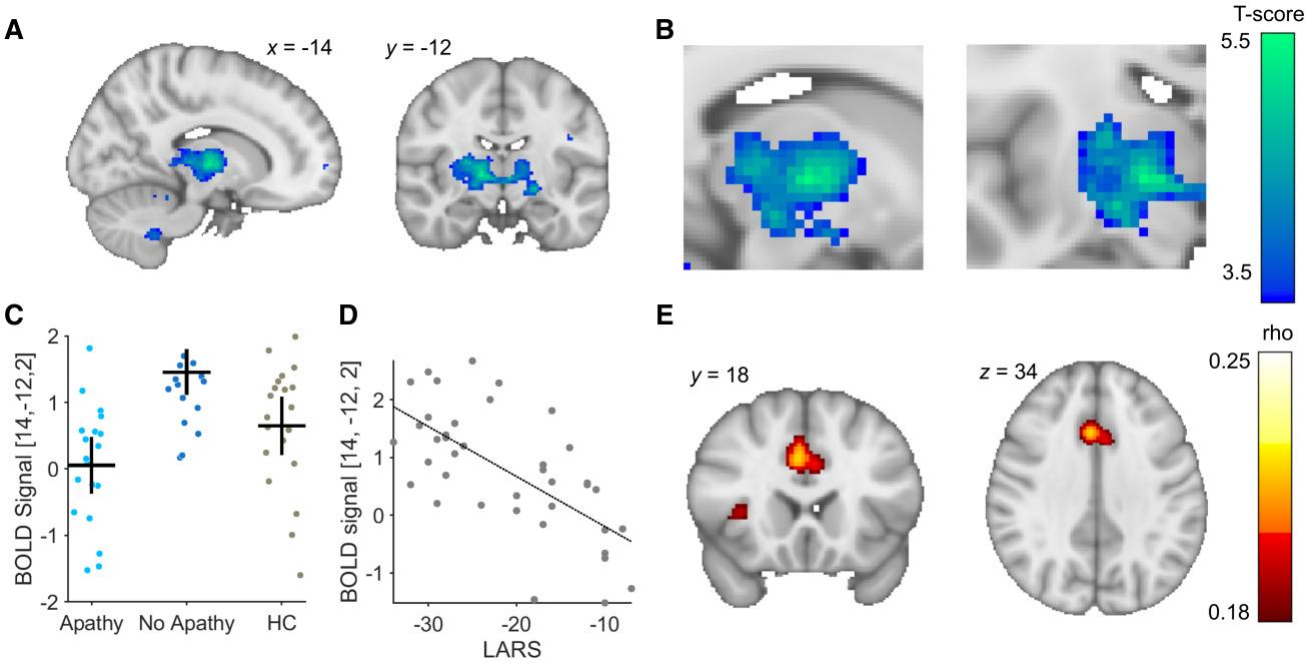
**Functional MRI BOLD contrast differences the decision time during exploratory choices.** (**A** and **B**) Peak contrast difference in activation between the PD-apathy and PD no-apathy within the right thalamus at the point of decision-making during an exploratory choice. Blood oxygen level-dependent (BOLD) signal activations at this peak voxel plotted for each subject and group (**C**) and correlation with individual apathy severity (**D**). Functional connectivity analysis at this voxel using normative functional resting state connectivity showed peak correlation between the thalamus and dACC/bilateral AI (**E**). AI = anterior insula; dACC = dorsal anterior cingulate cortex; HC = healthy controls; LARS = Lille Apathy Rating Scale.

This absence of thalamic/midbrain and occipitoparietal activation in the PD-apathy group was verified by a contrast analysis comparing the PD-no apathy and PD-apathy groups (PD apathy < PD no apathy). Post-contrast analysis, clusters that survived whole-brain FWE correction were identified in the bilateral thalamus, bilateral pre-post central gyrus, intraparietal sulcus, bilateral cerebellum and calcarine cortex [peak voxels right TH: (14, 12, 2), *T* = 5.37, *P*_cluster FWE WB_ < 0.001, right PCG: (6, −32, −72), *T* = 4.67, *P*_cluster FWE WB_ < 0.001, left IPS: (26, −66, −28), *T* = 4.46, *P*_cluster FWE WB_ < 0.001, left cerebellum: (−10, −58, −44), *T* = 4.57, *P*_cluster FWE WB_ < 0.001 right CC: (26, −52, −4), *T* = 4.89, *P*_cluster FWE WB_ < 0.001] (Fig. 6 and Supplementary Table 8).

The activity at the decision time during explore choices was also found to correlate with apathy severity in the same right thalamic cluster identified in the between group contrast analysis [*k* = 27, peak voxel right TH: (14, −12, 2), *T* = 5.44 *P*_cluster FWE WB_ = 0.005] (Fig. 6D).

Finally, to identify the cortical functional connection between this thalamic voxel that was a predictor of the patient’s apathy severity, we used Neurosynth.^61^ This confirmed peak functional connectivity at of rho = 0.22 corresponding to dACC (3, 18, 36) and bilateral AI (35, 7, −1) and (−34, 5, −1), rho = 0.23 (false discovery rate corrected for multiple comparisons *P* < 0.05, Fig. 6E).

## Discussion

We assessed the mechanisms of demotivated behaviour characterized as apathy in PD, using computational modelling of decision-making behaviour combined with event-related fMRI. The core results of this study are 3-fold. First, patients with PD-apathy are less able to monitor and choose the best option when faced with outcome uncertainty. Second, they use an exploratory decision strategy, which is best explained by impairment of incorporation of the neural representation of an options value into decision-making. Third, a loss of compensatory neural circuits implicated in decision evaluation predicts whether apathy is manifest in PD or not.

Reward insensitivity as a core feature of PD-apathy has been observed in several previous studies.^16,26,27^ Our results extend this, whilst simultaneously addressing the question of where in the neuronal processes that leads to motivated (and demotivated) decisions, reward sensitivity is lost. We found behavioural evidence of reward insensitivity in apathetic patients in the Restless Bandit Task. The ability to choose the most rewarding option was a strong predictor of their apathetic status. As revealed from computational modelling, PD patients with apathy make decisions that are indifferent to the learnt expected value of the different options. In modelling terms, this was reflected by reductions in the reward sensitivity parameter, β. By multiplying the value estimate for each option learned from one trial to the next, low levels of β drive up the proportion of non-greedy, ‘exploratory’ choices to the lower value options. In isolation, this analysis does not explain where the insensitivity to reward occurs and, by extension, its degradation, or the disregard of this learnt neural value representation. We were motivated to study learning in this task because it requires high levels of cognitive demand in both monitoring and learning from the outcomes of actions. At a neural level, to track and identify best option relies on a reliable and constantly updated representation of the outcomes value (and presumably the relative longer running reward average to compare this too).

One explanation for our results, is therefore, a failure of encoding the value of the chosen option within the brain.^62,63^ In non-human primates, neural firing rates correlate with the outcome received within the vmPFC.^64^ We reproduced the fMRI correlate of this outcome encoding described previously in our age-matched healthy control group in these regions. While this signal was blunted in both PD groups, it wasn’t a clear discriminator of their apathetic status. Caution is required in interpreting this signal, as this was less robust in the control group than that previously reported. This may be a reflection of the smaller sample sizes in our groups but is equally likely to reflect a disease- and age-related prefrontal dopaminergic denervation.^65,66^ Combining both the PD no-apathy with the healthy control group, we were able to demonstrate significant blunting of this signal in the PD-apathy patients. Therefore, impairment in the encoding of a choices outcome is a feature of PD-apathy, which is additional to that which occurs as a consequence of PD.^29^ However, as impairment of this signal did not correlate with an individual’s apathy severity, we would argue that this is unlikely, in isolation, to be the primary explanation for their demotivation.

At the decision time, we reproduced previously described fMRI correlate of exploration indexed by cortical activations including frontal pole, IPS, AI and preSMA/dACC in all three groups. Exploitative choices activated vmPFC/subgenual cingulate regions in the age-matched control group only. These regions have been implicated in the encoding of the expected value of chosen options.^67-69^ Despite no equivalent activation in both patient groups, paralleling the disease-related blunting of the outcome signal, we were not able to demonstrate any between group differences. In studies of younger controls, exploitation activates a more extensive set of regions (lateral OFC, posterior cingulate, hippocampus and precuneus^46,47,59,60^), which overlap with those of the default mode network (DMN).^70^ Age- and disease-related decline in the DMN signal in PFC may explain the absence of a significant between group difference as a consequence of reduced signal to noise.^71,72^

The overlap between activations during exploitation with regions in the DMN have led to the view of exploitation as a decision choice with lower cognitive and attentional demands.^73^ Exploration activates regions whose functions are synonymous with cognitive control. For example, the AI/dACC form nodes of the salience network, which heightens the detection of behaviourally relevant stimuli, response selection and conflict monitoring.^74^ The IPS is thought to serve as an interface between prefrontal regions and motor output, initiating the behaviour necessary to explore alternative actions.^46,75^ Finally, the frontal pole tracts the uncertainty of unsampled options to trigger switches from exploitative to exploratory behaviour.^60,76^ Recruiting these regions may explain why learning (and learning rates) increase during exploration.^38,77^ Why then, do our apathetic patients appear to perform worse in our task? We would argue that despite classification of these choices as exploratory, they are not analogous to exploration that occurs in health and do not reflect heightened cognitive control. First, exploration is costly.^21,78^ Intuitively, using a decision strategy that comes with additional cognitive cost is at odds with a clinical syndrome where novelty seeking and the motivation to undertake voluntary self-generated acts is diminished.^52^ Importantly, our finding that apathy severity was predicted by levels of random, but not directed exploration is consistent with both these clinical features and impaired task performance, as directed exploration relies upon working memory^55,79^ and aims to actively update information about the environment when uncertainty is greatest.^11,80-82^

Second, we found no difference in cortical activations during exploration in the PD-apathy and healthy control groups or additional activity in PD-apathy that was not present in the non-apathetic PD patients. This would argue against exploration being actively driven by a gain of neural function in which one or more brain regions is actively promoting this behaviour. This, for example, might be driven by uncertainty about the environment,^51^ or greater sensitivity to opportunity cost.^83^

We would argue that rather than being an active process to either seek out or learn new information, the shift from exploitation to exploration in PD-apathy, is a pathological signature of increased decision noise. Indeterminate, random selection rules are efficient strategies for exploration and are necessary for optimal adaptive choice.^55,84^ Suboptimal, ‘non-greedy’, random choices are a feature of normal decision-making in health and are thought to arise from limitations in the brain encoding of a decision’s value.^56,85,86^ By degrading the precision of outcome encoding or the encoding of an action’s expected value, prefrontal dopaminergic denervation could explain increased decision noise in PD.^87,88^ This loss of outcome and/or value encoding occurs independently of whether or not the patient has apathy.^29^

We interpret the finding of the increased activation of the explore circuit in PD-no apathy group as evidence for a compensatory mechanism that may protect against the manifestation of apathy. In support of this was the finding that the peak contrast difference between the PD groups correlated with individual apathy severity (LARS score). A compensatory mechanism is also more likely given that there was no difference between the PD-apathy and healthy control group activations, but increased activity in these regions was observed in the PD-no apathy group relative to the healthy controls. Connectivity between the medial and anterior thalamic nuclei and dACC positively correlates with enhanced cognitive performance and goal-directed behaviour in uncertainty.^89,90^ Additional recruitment of this circuit could preserve exploitation in our PD no-apathy group as this included cortical areas of the salience network and thalamus with peak functional connectivity with the dACC.

Alternatively, the same over-recruitment of these regions during exploration can also be viewed as an equivalent deactivation in the same regions during exploitation. During exploration, neural firing in PFC adopts a state transition into an indeterminate, disorganized and non-coding state.^91^ It may become imperative, when the brain loses the ability to encode value or outcome with precision, to suppress activity in circuits that may naturally augment additional neural decision noise. By over-suppressing activity in these (explore) regions during exploit decisions, this would limit their influence to promote decision noise being expressed into behaviour. This would support the possibility that in PD-apathy, the combination of both a loss of reward encoding and loss of mechanisms that override decision noise leads to demotivated behaviour. In our apathetic patients, the behavioural expression of this combined loss-of-function is manifest in decisions to explore rather than exploit.

### Study limitations

Could a simpler explanation of demotivated task engagement explain the behaviour of our apathy patients? For example, heightened exploration might be explained by an apathy-related indifference to task performance to allocating cognitive costs. Against this is that we found no clear difference in either the decision time or proportion of missed trials in the PD-apathy and no apathy groups. Furthermore, at the decision time, explore choices in the apathy group activated PFC regions including dACC and DLPFC, regions whose activation indexes engagement with task complexity^92^ and working memory.^93^ Their activation to levels comparable to that seen in the control group would make a deficiency in the allocation of cognitive control an unlikely explanation for their poorer task performance. Demotivation leading to task disengagement may also arise through heightened sensitivity to effort costs.^25^ Against this explanation, is that lower effort decision strategies, such as preservation, were less likely in our apathy patients.^94^ We would argue that the observed reduction in the proportion of perseverative choices is not consistent with a strategy minimizing the costs of cognitive control or an indifference to allocate effort or engage in the task.

It is important to emphasize that our results are unlikely to be generalizable to all phenotypes of apathy in PD. Consistent with previous studies,^26^ our patient’s exhibited the most commonly described subtype of PD apathy, one dominated by demotivation towards action initiation (Supplementary Table 1). Different neurobiological mechanisms could underpin apathy in patients with more emotional subtypes, apathy associated with comorbid cognitive impairment or, indeed, postoperative apathy in the context of deep brain stimulation.^95^

## Conclusions and future predictions

Allowing for these limitations, our results agree with the view that apathy in PD is unlikely to arise from a single loss of brain circuit function.^19,25,42^ Conceivably, the first step towards symptomatic manifestation of apathy is a loss of precision in stimulus value encoding,^96^ related to loss of prefrontal dopaminergic projections.^29,97^ We predict that the expression of demotivated behaviour in PD-apathy arises from secondary loss of compensation using neural decision circuits that can overcome limitations in value encoding. Obvious candidates include noradrenergic systems, which are proposed to regulate opponency of the DMN and salience network.^98^ Noradrenergic reuptake inhibitors are also actively considered as therapy for PD-apathy.^45^ Deficiencies in serotonin transporter expression in the dACC correlate with the severity of PD-apathy^41^ and its restoration to this area reverses apathy.^42^ As serotonin promotes choice persistence,^32^ upregulation of serotonergic systems could be an explanation for how our non-apathy group could continue to exploit, despite impaired encoding of a decision outcomes. A ‘double-hit’ phenomenon would be consistent with recent longitudinal imaging by Morris *et al*.,^99^ where loss of functional connectivity between dACC and ventral striatum in non-apathetic PD patients preceeded the clinical expression of a demotivated state.

Combining computational modelling of behaviour with neuroimaging of specific neuro-modulatory circuits may answer these predictions. New treatment targets identified with this approach should aim to augment neural circuit compensation identified in this study and protect from the manifestation of apathy in PD. Targeted neuromodulation of regions within this network, could represent a treatment intervention worthy of future investigation.

## Supplementary Material

awae025_Supplementary_Data

## Data Availability

All behavioural data, fMRI T-maps and code used for model fitting are available from https://osf.io/h3e5s/.
